# Transgenerational of Oxidative Damage Induced by Prenatal Ethanol Exposure on Spatial Learning/Memory and BDNF in the of Male Rats

**DOI:** 10.1016/j.ibneur.2024.09.001

**Published:** 2024-10-28

**Authors:** Mousa Shaabani Ghahremanlo, Vida Hojati, Gholamhassan Vaezi, Shahram Sharafi

**Affiliations:** Department of Biology, Damghan Branch, Islamic Azad University, Damghan, Iran

**Keywords:** Ethanol, Oxidative Stress, Brain-Derived Growth Factor, Spatial Learning and Memory, Transgenerational Impact, Rat

## Abstract

Alcohol consumption during pregnancy harms fetal development, leading to various physical and behavioral issues. This study investigates how prenatal ethanol exposure triggers oxidative stress (OS) and affects neurotrophic factors (NTFs), particularly brain-derived growth factor (BDNF) gene expression in the hippocampus, influencing learning and memory decline across two generations of male offspring from ethanol-exposed female rats. A rat model of fetal alcohol spectrum disorder (FASD) was initially generated to reflect on the deficits in the first generation, and then those transmitted via the male germline to the unexposed male ones. The pregnant rats were thus divided into four groups, namely, the control group (CTRL) receiving only distilled water (DW), and three groups being exposed to ethanol (20 %, 4.5 g/kg) by oral gavage, during the first 10-day gestation (FG), the second 10-day gestation (SG), and the entire gestation (EG) periods. Subsequent Morris water maze (MWM) tests on male offspring revealed spatial learning deficits during the second and entire gestational periods in both generations. Analysis of antioxidant enzyme activity including glutathione peroxidase (GPx), superoxide dismutase (SOD), and malondialdehyde (MDA), and BDNF gene expression in the hippocampus further highlighted the impacts of prenatal ethanol exposure. The study results demonstrated that prenatal ethanol exposure caused spatial learning/memory deficits during the SG and EG, altered antioxidant enzyme activity, and reduced BDNF gene expression in both generations. The findings underscore the role of OS in developmental and behavioral issues in FASD rat models and suggest that lasting transgenerational effects in the second generation may stem from alcohol-induced changes.

## 1 Introduction

Following the increased use of alcohol in various societies, its harmful effects are gradually being revealed. Extensive research is thus being conducted on the negative impacts of this drink. To date, fetal alcohol spectrum disorder (FASD) has been detected in children with prenatal ethanol exposure, presenting with the primary symptoms of delayed growth, facial birth deformities, and mental retardation (Abel and Hannigan, 1995). Strong experimental evidence further suggests that even low amounts of ethanol may have negative effects on the brain during normal fetal development (Everett et al., 2012). Recent findings propose that exposure to alcohol by the mother induces behavioral and structural modifications in future generations, as reported by Yohn and colleagues in 2015 (Yohn et al., 2015). In this respect, the hippocampus is a critical target for the toxic effects of ethanol in the course of brain development. Furthermore, spatial learning and memory impairments are prevalent symptoms observed in animals exposed to ethanol during fetal development, as substantiated by both experimental and clinical research (Streissguth et al., 1994, Sulik et al., 1981). The effects of alcohol on the hippocampus vary depending on the developmental stages, potentially inducing numerous changes during gestation, adolescence, and adulthood (Alfonso-Loeches and Guerri, 2011). Extensive research has elucidated the time-specific ramifications of ethanol exposure (Hamilton et al., 2010, Patten et al., 2014, Gianoulakis, 1990), with disparities in intensity apparent across various phases of brain maturation. In spite of this, some investigations on various ethanol exposure times have established no safe time to drink while being pregnant (May et al., 2013). Many mechanisms have been additionally proposed so far to explain the neurotoxicity caused by ethanol consumption. As an illustration, alcohol metabolism can instigate oxidative stress within cells by augmenting the levels of reactive oxygen species (ROS) (Guerri et al., 1994), and it also possesses the capability to modulate the function of endogenous antioxidant enzymes through oxidation (Brocardo et al., 2011). Once the levels of ROS within cells reach an excessively high threshold, the endogenous protective mechanisms are unable to effectively mitigate oxidative damage over an extended duration (Tran et al., 2005). Free radicals exert detrimental effects on mitochondria and other subcellular organelles by facilitating the oxidation of nucleic acids, proteins, inducing DNA fragmentation, and promoting lipid peroxidation (Cabral-Costa and Kowaltowski, 2020, Singh et al., 2022). OS triggers apoptosis through the mitochondria-mediated pathway, where the proapoptotic protein Bax induces mitochondrial membrane breakdown and cytochrome c release (Mo et al., 2017), leading to the activation of caspase-3 via interaction with pro-caspase 9 in the brain that triggers the onset of apoptosis (Young et al., 2003).Neuronal defects and cognitive impairments resulting from ethanol exposure are linked to disturbances in neurotrophic factor (NTF) support, impacting neuronal survival and protective mechanisms. Studies show that alcohol exposure during brain development reduces BDNF gene expression, altering its receptor, tropomyosin receptor kinase B (TrkB) (Climent et al., 2002). Some investigations have correspondingly found a link between OS and the BDNF gene expression as well as their involvement in multiple spatial learning/memory deficits (Beheshti et al., 2019, Molteni et al., 2004). In a study on ethanol exposure during the gestational days 5–20, the reduced BDNF gene expression and mRNA levels had been thus detected in the cortex and hippocampus of rat models (Feng et al., 2005), while other investigations had reported an upward trend or no changes in the BDNF mRNA expression in this respect (Caldwell et al., 2008). Accordingly, the effects of ethanol exposure on the hippocampal BDNF gene expression seem to be unpredictable. Some inconsistencies in the study results can be further related to the dose, administration time, exposure time, as well as method of ethanol administration, such as inhalation, intraperitoneal (IP) injection, or gavage. Remarkably, the alterations induced by ethanol exposure can exhibit heritable traits, being transmitted across generations and affecting gene expression in individuals who have never been directly exposed to ethanol (Govorko et al., 2012). Data from multiple sources have additionally identified an augmented susceptibility to ethanol and heightened alcohol consumption in subsequent generations (Nizhnikov et al., 2016, Mead and Sarkar, 2014).

In line with this perspective, Koabel et al. (2021) reported a higher probability of anxious behavior and increased alcohol consumption in the second generation, whose grandparents had been previously exposed to ethanol (Koabel et al., 2021). In the present study, the main objective was to evaluate the transgenerational effects of different prenatal ethanol exposure times (namely, the first and second 10-day as well as entire pregnancy periods). One of the key hypotheses driving this research is that prenatal ethanol exposure-induced OS disrupts the delicate balance of oxidative processes in the developing brain, leading to alterations in BDNF gene expression in the hippocampus. These changes, in turn, may contribute to deficits in learning and memory observed in the offspring. By investigating these relationships across two generations of male rats, the study aims to provide valuable insights into the lasting effects of maternal ethanol exposure on neurodevelopment and behavior.

## 2 Materials and methods

Adult male and female Wistar rats, weighing 200–180 g, were purchased from the Razi Vaccine and Serum Research Institute, Karaj, Iran. Then, they were transferred to the animal house, kept in a cage with 12 hours of light and 12 hours of darkness, with a controlled environmental temperature (20–22 °C) and free access to food and water. All the experiments were carried out and authorized by the Research Ethics Committee affiliated to Islamic Azad University (IAU), Damghan Branch, Damghan, Iran, in accordance with the international laws of ethics and protection of laboratory animals, with the code no. IR.IAU.DAMGHAN.REC.1401.016.

The pregnant rats (F0) were accordingly divided into four groups of 5 rats. the control group (CTRL) receiving only distilled water (DW) and three other groups being exposed to ethanol (20 % v/v, 4.5 g/kg) by oral gavage in the morning (10 a.m.). The ethanol groups were divided to three groups based on the 21-day gestation period of rats. This division entailed exposing rats to ethanol during the first 10-day gestation (FG), the second 10-day gestation (SG), and the entire gestation (EG) periods to explore varying effects across these different periods. To create the second filial generation (F2) subset, the first filial generation (F1) males were mated with the females in the CTRL group. This study is solely conducted on male offspring, the male germline was used to produce subsequent generations following a single generation ethanol exposure. In addition, Studies were also carried out on male rats in the second generation. The total numbers of males and females used in the entire study are presented in Table 1.

**Table 1:** Total numbers of animal used in the study.

	♂		♀	
F0	CTRL: 20	ETH: 0	CTRL: 5	ETH: 15
F1	CTRL: 19^a^	ETH: 57^b^	CTRL: 20	ETH: 0
F2	CTRL: 14	ETH: 42	CTRL: 0	ETH: 0

a : 19 males including 5 for mating + 14 for behavioral and biochemical studies.

b : 57 males including15 for mating + 42 for behavioral and biochemical studies.

**Fig. 1:**
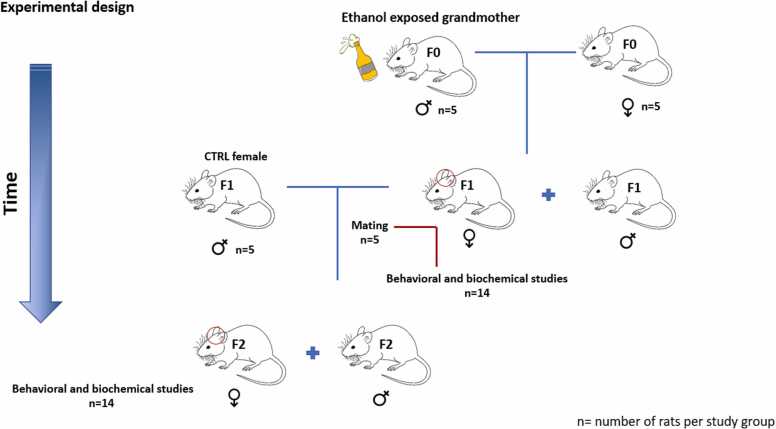
Experimental design.

The litter size was also planned to have approximately 12 offspring (namely, 7–8 males and 4–5 females). On the postnatal day 21, a total of 14 male offspring in the F1 and the F2 were separated. Some offspring were included in the analysis set as 1 per litter for the biochemical tests and 1–2 per litter for the behavioral ones. In total, 5 male rats from each group were anesthetized with ketamine and xylazine, afterward the hippocampus was removed and stored in a −70 °C until the biochemical and molecular studies were performed. The other nine male offspring were further subjected to behavioral tests 24 hours later to measure spatial learning/memory, using the Morris water maze (MWM) test (Fig. 1).

### 2.1 MWM test

The Morris water maze test is employed to examine the hippocampal-related learning and retention of spatial memory over the long term. Therefore, in this study MWM test used to study spatial memory in rats exposed to ethanol from two successive generations. The MWM task consist of two parts: four days of training, to evaluate learning ability and probe test, to evaluate spatial memory 24 h after training days. It was performed as described before (Toosi et al., 2019), 24 days following birth to investigate spatial learning and memory. The water maze was made up of a black pool containing water. The tank was divided into four quadrants. The detachable platform was located below the water's surface. During the probe test, the rats had to remember the location of the platform in a variety of external cues. Each rat was allowed to swim to discover the platform during trial days. The time it took animals to move up on the hidden platform was recorded as escape latency, and the distance traveled to reach the platform was recorded as distance moved. In order to determine the memory of the animals, probe test was performed. The platform was eliminated 24 hours after the last training sessions and the rat was placed into the water. Time spent in the "target quadrant" (the quadrant that previously contained the platform) was measured, and time to reach in the zone that initially includes the platform was noted as escape location latency. A video camera placed on the ceiling above the core of the pool recorded all procedures, and an automated monitoring device tracked the animal's following behavior (latency, distance, and swim speed) (Ethovision; Noldus IT, the Netherlands).

### 2.2 Malondialdehyde (MDA) assessment

The amount of MDA produced is dependent on the amount of unsaturated fatty acids broken down and separated. As a consequence, measuring MDA is an appropriate index for lipid peroxidation. In this method, MDA reacts with thiobarbituric acid (TBA) in an acidic pH to produce a pink color. The spectrophotometric was used to measure the absorbance of the reaction mixture at 535 nm (Shafahi et al., 2018).

### 2.3 Assessment of the anti-oxidative enzyme’s activity

#### 2.3.1 SOD activity measurement

SOD activity was measured based on inhibition of photochemical reduction of nitroblue terazolium chloride (NBT) in the samples. The mixed solution was consisting of the following ingredients: K-phosphate buffer (210 M at pH = 7.2), methionine (2.2 M), Riboflavin (2.2 M) and NBT (82.5 μM). A spectrophotometer was used to measure the absorbance of different groups at 560 nm. One unit of SOD (U) was defined as the amount of enzyme that prevents NBT reduction by 50 %. The activity was measured in terms of units per mg of protein (Bagheri et al., 2022).

#### 2.3.2 GPx Activity measurement

GPx activity was measured using tert-butyl hydroperoxide as a substrate and NADPH usage by glutathione reductase for 2 minutes at 37°C and 340 nm.Reduced glutathione (2 mM), glutathione reductase enzyme (0.15 u/ml), sodium azide (0.4 mM), tert-butyl hydroperoxide (0.5 mM), NADPH (1 mM), and K-phosphate buffer (10 mM at pH = 7.2) were all prepared for the reaction (Bagheri et al., 2015).

### 2.4 Semiquantitative reverse transcription-polymerase chain reaction (RT-PCR) analysis

Extracted RNA was prepared from hippocampus using TriZol reagent. Using spectrophotometer, the optical density at A260/280 ratio (1.8–2.0 was considered pure) was measured and gel electrophoresis were used to determine RNA quality. cDNA synthesis was performed in compliance with the manufacturer's instructions for the PrimeScript TM 1st Strand cDNA Synthesis Kit (Takara, Japan). A thermalcycler was applied to perform PCR (Eppendorf, Germany). PCR products were electrophoresed using agarose gel. The BDNF gene was relative quantified using image J software, and the PCR results were normalized with the GAPDH mRNA expression. Table 1 provides the forward and reverse primer sequences used in the study. The RTq-PCR products were 405 bp for BDNF, and 203 bp for GAPDH. Table 2

**Table 2:** Specifications of the primers used in the research.

**Primer**	**Sequence**	**Primer size (bp)**	**Product size (bp)**
GAPDH-F	5'-TGACATCAAGAAGGTGGTGAA−3′	21	203
GAPDH-R	5'-CCCTGTTGCTGTAGGCGTATT−3′	21	
BDNF-F (Soltanian et al., 2013)	5'-GCCCAACGAAGAAAACCATA−3′	20	405
BDNF-R	5'-GATTGGGTAGTTCGGCATTG−3′	20	

## 3 Statistical analysis

Graph Pad prism 9 software was used to analyze the data, which included two-way and one-way ANOVA. Tukey's post hoc test was applied to the data in groups. The results are presented as mean standard error of the mean (SEM) and are considered significant at P≤0.05.

Additionally, Shapiro-Wilk test was performed to test normality of data set.

## 4 Results

### 4.1 Impacts of prenatal ethanol exposure on spatial learning/memory in F1 male rat offspring

**Fig. 2:**
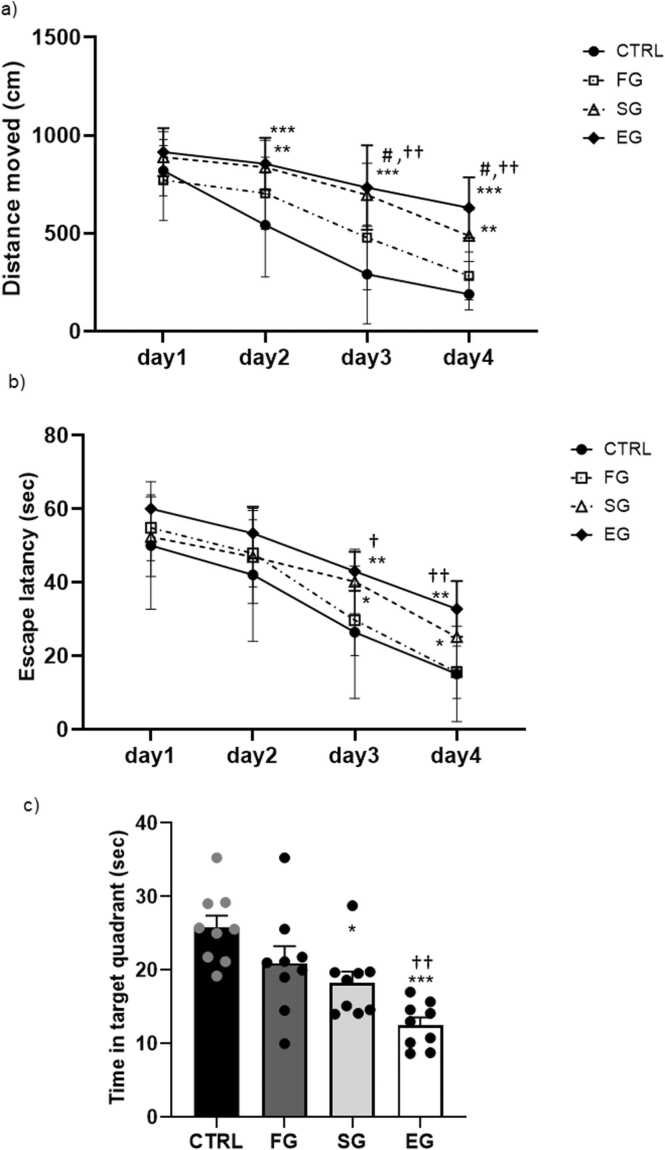
**Alcohol exposure impacts on the performance of spatial memory in the MWM test (F1). Distance moved (a), average scape latency (b), time in target quadrant (c) in training days.** a) The distance traveled notably increased in the SG and EG groups compared to the CTRL group from days 2–4, but not during the FG period. Significant differences were observed between the FG group and the other groups on days 3 and 4. b) Escape latency notably increased in the SG and EG groups on days 3 and 4 compared to the CTRL group. Within-group analysis also revealed significant differences between the FG and EG groups on days 3 and 4. c) The SG and EG groups spent less time in the target zone than the CTRL group, with a significant decrease observed in the EG group compared to the FG group. ***P<0.001, **P<0.01, *P<0.05 compared to CTRL. #P<0.05 FG vs SG group. ††P<0.01, †P<0.05 FG vs EG group.

The MWM test results were assessed to learn about the spatial learning/memory ability of the animals (n= 9 in each group). The repeated measures two-way analysis of variance (ANOVA) results of the distance traveled during this test showed the main effects of days (F_3,24_=41.63, P<0.001), groups (F_3,24_=21.09, P<0.001), and group×day (F_9,27_=2.144, P<0.03). As evidenced by Tukey’s post-hoc test outcomes, the total distance traveled significantly augmented in the SG and EG groups compared with that in the CTRL group on days 2–4 (P<0.01, P<0.001, respectively) and not in the FG period (Fig. 2a). The comparison between the study groups also demonstrated a significant difference between the FG group with two other ones exposed on days 3 and 4 (P<0.05, P<0.01, respectively).

The data analysis of the escape latency parameter in the F1 male offspring (Fig. 2b) further confirmed the significant effect of days (F_3,24_=45.56, P<0.001), groups (F_3,24_=10.89, P<0.001), and no interaction in group×day (F_9,27_=0.929, P<0.505). Moreover, the Tukey’s post-hoc test outcomes confirmed a significant rise in the escape latency in the SG and EG groups on days 3 and 4 (P<0.05, P<0.01respectively) than in the CTRL group. The within-group comparison additionally evidenced a significant difference between the FG and EG groups on days 3 (P<0.05) and 4 (P<0.01). Although no significant variances were noted between the FG and SG groups regarding the time taken to locate the platform, the control group exhibited superior learning and memory retention in the MWM compared to the experimental group. This was evidenced by shorter latencies and path lengths in the control group.

The probe test was correspondingly performed to evaluate spatial memory retention, and then record the time spent in the target quadrant (Fig. 2c). The one-way ANOVA results accordingly demonstrated a significant difference between the study groups (F_3,32_=10.49, P<0.001). In this line, the SG (P<0.05) and EG (P<0.001) groups spent less time in the target zone compared with the CTRL group, and a significant decrease was detected in the EG group as compared with the FG group (P<0.01). The ethanol groups (SG and EG) exhibited prolonged latency in finding the original platform location, indicating impaired memory retrieval and spatial navigation abilities compared to CTRL group.

### 4.2 Impacts of prenatal ethanol exposure on OS markers in F1 male rat offspring

**Fig. 3:**
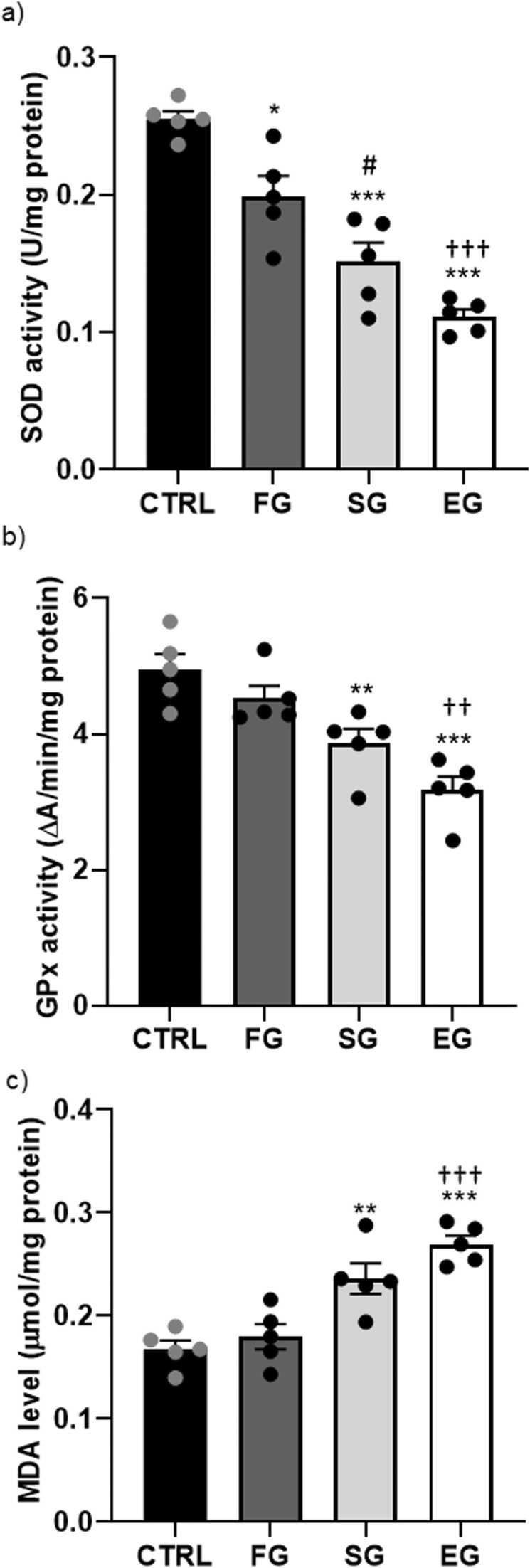
**Alcohol exposure impacts on the oxidative markers of the F1 male rat pups. SOD activity (a), GPx activity, MDA level (c) in the hippocampus.** a) SOD activity notably decreased in the FG group compared to the CTRL group. There was a significant difference in SOD activity between the FG group and the SG and EG groups. b) GPx activity significantly decreased in the EG group compared to the FG group.c) The hippocampal MDA level was significantly higher in the SG and EG groups than in the CTRL group. Notably, the MDA level differed significantly between the FG and EG groups. ***P<0.001, **P<0.01, *P<0.05 compared to CTRL. #P<0.05 FG vs SG group. †††P<0.001, ††P<0.01 FG vs EG group.

As per the one-way ANOVA outputs, a significant difference was observed between the study groups in terms of the SOD (F_3,16_=32.33, P<0.001) and GPx (F_3,16_=13.86, P<0.001) levels (n=5 male rats in each group). The SOD and GPx activity in the SG and EG groups were notably lower than those in the CTRL group (P<0.001), but the SOD activity showed a significant decrease in the FG group compared with the CTRL group (Fig. 3a, b). Comparing the study groups exposed to ethanol also established a significant difference in the SOD activity between the FG and the SG and EG groups (P<0.05, P<0.001, respectively). Furthermore, the GPx activity significantly reduced in the EG compared with the FG group (P<0.01). Moreover, there was a significant change in the MDA level (F_3,16_=17.84, P<0.001). The post-hoc comparison additionally showed that the hippocampal MDA level (Fig. 3c) significantly elevated in the SG and EG groups than in the CTRL one (P<0.01, P<0.001, respectively). Of note, the MDA level was significantly different in the FG and EG groups (P<0.001).

### 4.3 Impacts of prenatal ethanol exposure on BDNF mRNA expression in F1 male rat offspring

**Fig. 4:**
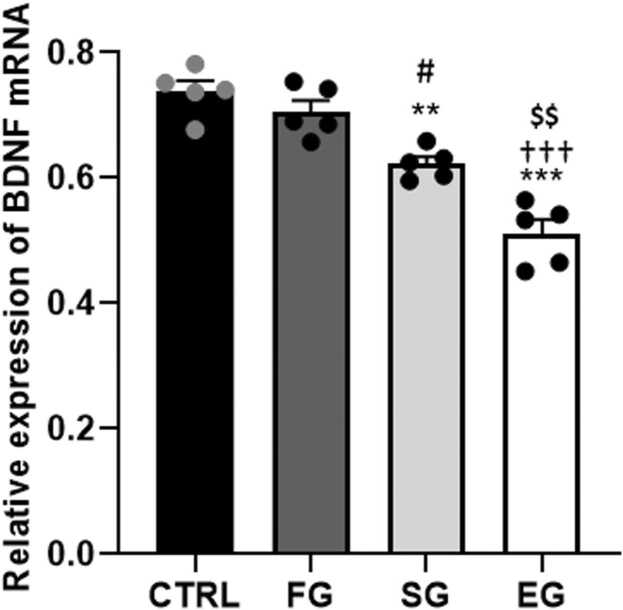
**Alcohol exposure impacts on the BDNF mRNA expression of the F1 male rat pups in the hippocampus.** BDNF expression was notably reduced in the SG and EG animals compared to the CTRL group. The decrease was significant in both SG and EG groups compared to the FG group. Additionally, BDNF mRNA expression significantly dropped in the EG group compared to the SG group. ***P<0.001, **P<0.01 compared to CTRL. #P<0.05 FG vs SG group. †††P<0.001 FG vs EG group. $$P<0.01 SG vs EG group.

The one-way ANOVA outcomes of the BDNF mRNA expression showed a significant difference between all study groups (F_3,16_=32.9, P<0.001). Compared with the CTRL group, the gene expression in the SG and EG animals significantly downregulated (P<0.01, P<0.001, respectively), and the reduction of the BDNF gene expression was significant in both SG (P<0.05) and EG (P<0.001) groups compared with the FG group. On the other hand, the BDNF mRNA expression significantly declined in the EG group than in the SG group (P<0.01) (Fig. 4).

### 4.4 Impacts of prenatal ethanol exposure on spatial learning/memory in F2 male rat offspring

**Fig. 5:**
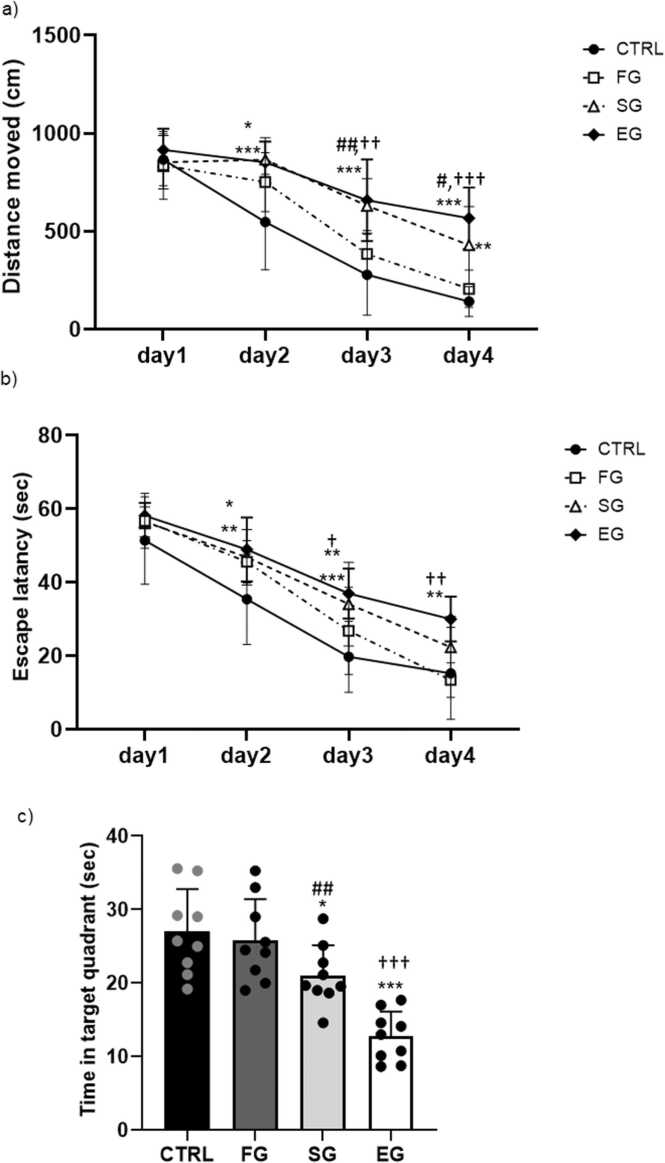
**Alcohol exposure impacts on the performance of the spatial memory in the MWM test (F2) Distance moved (a), average scape latency (b), time in target quadrant (c).** a) On day 2, total distance traveled significantly increased in the FG, SG, and EG groups compared to the CTRL group. Across days 3 and 4, there was a notable rise in distance traveled in the SG and EG groups compared to the CTRL group. Significant differences were also observed between the FG and SG groups as well as between the FG and EG groups on days 3 and 4. b) Both the SG and EG groups exhibited a significant increase in escape latency on days 2 and 3 compared to the CTRL group. On day 4, only the EG group showed a significant increase compared to the CTRL group. Additionally, there was a significant difference in escape latency between the FG and EG groups on days 3 and 4. c) The EG group spent less time in the target quadrant than the CTRL group and significantly less time than the FG and SG groups. ***P<0.001, **P<0.01, *P<0.05 compared to CTRL. ##P<0.01 FG vs SG group. †††P<0.001, ††P<0.01, †P<0.05 FG vs EG group.

The contrasting behaviors observed between the control and ethanol groups suggest a potential link between ethanol exposure and impaired spatial learning processes in F2 males. According to the repeated measures two-way ANOVA results of the distance traveled, the main effects of days (F_3,21_=129.4, P<0.001), groups (F_3,21_=20.4, P<0.001), and group×day (F_9,63_=3.18, P<0.003) were established (n=8). On day 2, the total distance traveled significantly increased in the FG (P<0.05), SG, and EG (P<0.001) groups compared with that in the CTRL group. As well, a significant upward trend was observed in the SG (P<0.001, P<0.01, respectively) and EG (P<0.001) groups during days 3 and 4 than that in the CTRL group (Fig. 5a). The comparison between the groups exposed to ethanol also established a significant change on days 3 and 4 between the FG and SG (P<0.01, P<0.05, respectively) and FG and EG (P<0.01, P<0.001, respectively) groups.

Moreover, the escape latency parameter in the F2 male offspring disclosed significant effects in days (F_3,21_=90.65, P<0.001), groups (F_3,21_=20.49, P<0.001), and no interaction in group×day (F_9,63_=1.33, P<0.240). In the SG (P<0.05, P<0.01, respectively) and EG (P<0.01, P<0.001, respectively) groups, there was also a significant rise in the escape latency parameter on days 2 and 3 as compared with that in the CTRL group. On day 4, only the EG group showed a significant increase in comparison with the CTRL group (P<0.01) (Fig. 5b). Moreover, the escape latency indicated a significant difference between the FG and EG groups on days 3 and 4 (P<0.05, P<0.01, respectively).

According to the probe test, the time spent in the target quadrant was recorded. The one-way ANOVA outcomes accordingly proved a significant difference between the study groups (F_3,32_=16.11, P<0.001). The EG group also spent less time in the target quadrant compared with the CTRL group (P<0.001) (Fig. 5c). Likewise, the within-group comparison showed that the EG group spent notably less time than the FG (P<0.001) and SG (P<0.01) groups. In contrast to the control group, the F2 males of ethanol-exposed rats displayed a less pronounced preference for the target quadrant during the probe trial. The ethanol group exhibited increased exploratory behavior in non-target quadrants, suggesting impaired spatial memory consolidation.

### 4.5 Impacts of prenatal ethanol exposure on OS markers in F2 male rat offspring

**Fig. 6:**
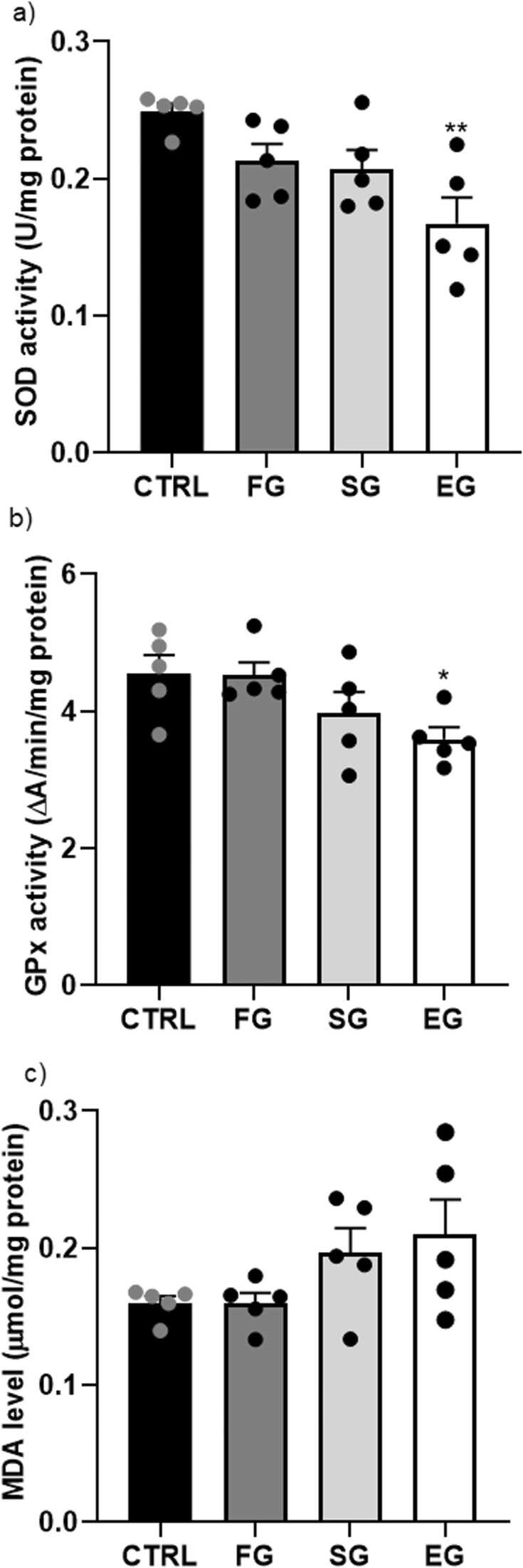
**Alcohol exposure impacts on the oxidative markers of the F2 male rat pups. SOD activity (a), GPx activity, MDA level (c) in the hippocampus.** a) SOD activity notably decreased in the EG group compared to the CTRL group, while b) GPx activity significantly declined compared to the CTRL group. c) There were no significant changes observed between the study groups regarding the MDA level of the F2 generation. **P<0.01, *P<0.05 compared to CTRL.

The one-way ANOVA outputs revealed a significant change in the SOD (F_3,16_=6.049, P<0.006) and GPx (F_3,16_=3.70, P<0.03) activity between the study groups (n=5 in each group). As established by Tukey’s post-hoc test results, the SOD activity significantly decreased in the EG (P<0.01) compared with that in the CTRL group (Fig. 6a). Moreover, the GPx activity remarkably declined in comparison with that in the CTRL group (P<0.05) (Fig. 6b). In general, no significant change was found between the study groups in terms of the MDA level of the F2 generation (F_3,16_=2.38, P=0.107) (Fig. 6c).

### 4.6 Impacts of prenatal ethanol exposure on BDNF mRNA expression in F2 male rat offspring

**Fig. 7:**
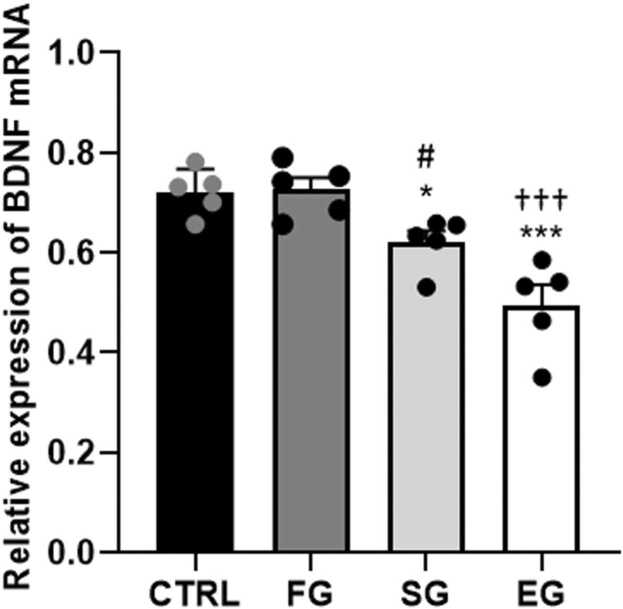
**Alcohol exposure impacts on the BDNF mRNA expression of the F2 male rat pups.** BDNF mRNA expression significantly decreased in the EG group compared to the CTRL group. it showed a significant reduction in the EG group compared to the FG and SG groups. ***P<0.001 compared to CTRL. #P<0.05 FG vs SG group. †††P<0.001 FG vs EG group.

A significant difference was observed between the study groups (F_3,16_=14.7, P<0.001) according to the one-way ANOVA results of the BDNF mRNA expression. The Tukey’s post-hoc test outcomes further indicated the gene expression only decreased significantly in the EG (P<0.001) than in the CTRL group (Fig. 7). Furthermore, the BDNF mRNA expression suggested a significant reduction in the EG compared with the FG (P<0.001) and SG (P<0.05) groups in the F2.

## 5 Disscusion

The study findings revealed that ethanol exposure and toxicity at various stages of pregnancy could induce OS in the brain, resulting in spatial learning and memory deficits in the later life of the F1. The severity of the impairments might thus differ according to the time and duration of the exposure. However, limited knowledge has been acquired thus far regarding the implications experienced by subsequent generations that were not exposed to adverse intrauterine conditions. Therefore, the current study aimed to explore the effects of prenatal ethanol exposure on the F2 male rat offspring, specifically focusing on spatial learning and memory deficits, as well as potential associations with oxidative damage in the hippocampus.

We measured the OS markers in the offspring's hippocampal tissues to uncover the significant role of OS as a mediator in the cognitive abnormalities associated with ethanol exposure. Furthermore, the MWM test along with the BDNF gene expression was assessed as the crucial components in hippocampal development and neuronal survival, as well as the basis of spatial learning and memory.

Based on previous research, ethanol exposure during normal fetal development could raise OS markers in the brains of the newborn or adult rats (Boschen and Klintsova, 2017, Virgolini et al., 2019). The oxidative damage observed in different animal models of FASD accordingly depended on the brain region, the time and pattern of ethanol consumption, alcohol dosage, the blood alcohol concentration, and the age at the time of the analysis (Brocardo et al., 2011, Bagheri et al., 2015, Mahdinia et al., 2021). Even so, there is no strong evidence that the effects of ethanol exposure during the trimesters differ significantly. The pattern of drinking and the developmental period of ethanol intake can thus result in a wide variety of effects on offspring, ranging from moderate to severe cognitive and behavioral disorders (Maier and West, 2001, Guerri et al., 2009, Cuzon Carlson et al., 2020). In this study, the induction of oxidative damage was confirmed by the evaluation of the OS markers in the F1 all through three time periods of gestation. The results correspondingly indicated that the MDA levels significantly elevated in the rats in the SG and EG groups exposed to ethanol. A significant decrease was further observed in the GPx and SOD activity in these groups as well. In this regard, many studies focusing on the role of oxidative stress in neurodegenerative disorders such as depression, Alzheimer’s disease, Parkinson disease and ischemic stroke (Bhatt et al., 2020, Huang et al., 2016, Orellana-Urzúa et al., 2020). As the central nervous system (CNS) also contained far fewer antioxidants than other tissues, the elevated levels of unsaturated fatty acids within the CNS render it more susceptible to the detrimental effects of free radicals. OS plays a detrimental role in neurodegeneration by causing damage to neural cells through free radical attacks. The toxic effects of ROS contribute to various harmful processes, including protein misfolding, activation of glial cells, disruption of mitochondrial function, and ultimately, cellular apoptosis (Fulda et al., 2010). The amount of LP could accordingly increase in the SG and EG groups, implying that the hippocampus might face greater damage during the SG than the FG. Additionally, augmenting the prenatal ethanol exposure length (namely, the total gestational days) could be efficient in enhancing toxicity induced by ethanol metabolism.

To evaluate the association between OS in the hippocampus and memory impairment, the qRT-PCR technique was exploited to examine the BDNF gene expression in the hippocampal tissue of male offspring over two generations. Of note, it is well known that the expression of neurotrophins and their receptors is typically regulated during the CNS development, and the accurate regulation seems critical for this process. Furthermore, neurotrophins and their receptors are mostly expressed in the hippocampus and cortex, which are the vital areas of neuronal plasticity related to spatial learning/memory (Chao, 2003). The interaction between BDNF and the TrkB receptor triggers a series of intracellular signaling pathways that lead to the phosphorylation and activation of the transcription factor known as cAMP response element binding protein (CREB). Once phosphorylated, CREB moves into the nucleus and binds to BDNF promoters, facilitating the transcription of BDNF. This process enhances BDNF expression, which is essential for neuronal survival, differentiation, and synaptic plasticity (Song et al., 2013, Pradhan et al., 2019). Indeed, the BDNF is a potent synaptic facilitator, whose deficiency leads to long-term potentiation of abnormalities as well as spatial learning/memory deficits, which can be then alleviated by administering exogenous BDNF (Boschen and Klintsova, 2017). In the present study, the BDNF mRNA expression significantly declined in the SG and EG groups of the F1. In this context, Kown (2013) had indicated that repeated treadmill exercise could improve cognitive impairments caused by restraint stress-induced OS and reduced BDNF levels (Kwon et al., 2013). According to previous research, this descending trend might play a key role in spatial learning and memory deficits in offspring (Alzoubi et al., 2013, Wang et al., 2020). The evaluations of the spatial learning/memory have further shown that alcohol intake in the SG as well EG might cause learning difficulties and poor spatial and cognitive memory in rat offspring. When comparing the CTRL group to the SG and EG groups, there was a noticeable increase in the time it took to find the platform and the distance traveled. Additionally, the time spent in the target quadrant significantly decreased in the SG and EG groups, as opposed to the CTRL group. These findings strongly suggest that the SG and EG groups exhibited deficits in spatial learning and memory. In agreement with the present report, many research has discovered that prenatal ethanol exposure can impair cognition and lead to lasting neurobehavioral impacts (Mahdinia et al., 2021, Marquardt and Brigman, 2016, Kodituwakku and Kodituwakku, 2014).

Most studies in the field of prenatal ethanol exposure have primarily focused on the negative effects on the F1 generation. However, research on alcohol exposure has also revealed the potential for multigenerational ethanol impacts that might be mediated by epigenetic changes found in the male germline of rats (Govorko et al., 2012). In this study, alcohol was given to pregnant rats to produce the F1, and then male F1 rats were mated with the CTRL females to produce the F2. All F1 male offspring studies were also conducted on the F2 ones. Based on the most recent investigations, maternal ethanol exposure might promote tissue-specific epigenetic changes that are transferred to the subsequent generations, thereby resulting in malfunction in non-treated offspring (Chen et al., 2013, Gangisetty et al., 2020). In the second part of the study, there were no significant modifications of the OS markers in any group except the EG group, and there were no significant differences in LP across all groups. In this context, the qRT-PCR results revealed a significant reduction in BDNF specifically in the EG group, which coincided with the spatial learning and memory deficits observed in this group.

A possible explanation for the outcomes might be that prenatal ethanol exposure could cause epigenetic changes in the regulatory genes of brain development, such as the DNA methylation, histone acetylation, etc., which play an important role in the abnormal neural development of FASD (Berkel and Pandey, 2017, Mahnke et al., 2017).

From another perspective, the epigenetic support for transgenerational environmental consequences can only be accepted if the effects of maternal exposures last for three generations and paternal exposures for two generations (Jirtle and Skinner, 2007). This rationale stems from the fact that a gestating female in the F0 generation directly exposes both F1 generation fetuses and their germ cells, which will produce the F2 generation.

In current study, the effects observed in the F1 and F2 generations attributable to direct exposure of ethanol and thus classified as parental effects. Considering that in the second part of our study we observed a significant reduction in antioxidant enzymes activity only in the EG group, we can conclude that the duration of ethanol exposure serves as a crucial factor leading to lasting changes and impairments in memory and learning trough reduction of BDNF expression. Another noteworthy observation is that in the F2 generation, while there was no significant change in antioxidant enzyme activity in the SG group, a substantial reduction in BDNF expression was observed. This indicates that maternal ethanol exposure, although not disrupting the antioxidative balance in the F2 generation, resulted in alterations in neurotrophic factor expression, ultimately leading to impairments in memory and learning.

However, an issue not addressed in this study was whether prenatal ethanol exposure could lead to epigenetic changes in the genes in the germline as well as its relationship with alcohol-induced cognitive impairments in the subsequent generations. Therefore, further experimental investigation is needed to address this possibility. Due to time constraints, this study was exclusively focused on male subjects. Nevertheless, for comparative purposes between the sexes, additional research involving both genders are deemed essential.

## 6 Conclusion

The study findings suggested the involvement of OS in producing developmental and behavioral abnormalities in a rat model of FASD. In addition, the F2 studies indicated that the problems associated with prenatal ethanol exposure could be inherited in offspring. However, further investigation is still required to demonstrate the epigenetic changes in the genes involved in the hippocampal development and memory formation to evaluate this hypothesis.

## Ethical approval

All applicable international and national guidelines for the care and use of animals were followed.

## Author contributions

M.Sh performed all experiments and wrote the manuscript. V.H designed the experiment and review the manuscript. All authors verified the final manuscript.

## Funding

This study was supported by 10.13039/501100002660Islamic Azad University of Damghan branch.

## CRediT authorship contribution statement

**Mousa Shaabani Ghahremanlo:** Project administration. **Shahram Sharafi:** Visualization, Validation. **Gholamhassan Vaezi:** Methodology. **Vida Hojati:** Project administration, Conceptualization.

## Declaration of Competing Interest

The authors declare that they have no known competing financial interests or personal relationships that could have appeared to influence the work reported in this paper.

## Data Availability

The data that support the findings of this study are available from the corresponding author, upon reasonable request.
